# The Local Government Board and Unqualified Practice

**Published:** 1909-06-05

**Authors:** 


### The Local Government Board and Unqualified Practice.

A circular has recently been issued from the Local Government Board offices to medical officers of health on the subject of unqualified practice. The President of the Board is desirous of obtaining definite information on the points raised by the resolution adopted by the General Medical Council calling for a Royal Commission to investigate the evils produced by the unrestricted practice of unqualified persons; and in this circular he asks for replies to two specific questions. First, whether unqualified practice is increasing in the M.O.H.'s district; secondly, the effects of such practice on the public health. We notice that a correspondent of the *British Medical Journal*, who received one of these circulars, filled it up by saying that there is no unqualified practice in his district except that of chemists, who both prescribe for illnesses and also treat injuries by the application of dressings, etc. He added that he has known cases in which the delay thus caused has been not only serious, but even fatal.

Now this correspondent clearly shows that the practice of what is called "counter prescribing" on the part of chemists is in his opinion the most dangerous or at least the most prevalent form of unqualified medical practice in his neighbourhood. Whether he is a "whole time" or "part time" medical officer of health is not stated; but this point is one which the defenders of "counter prescribing" (whose views are often to be read in their trade journals) are likely to raise. For if this particular M.O.H. should be known to be engaged in general dispensing practice as well as in the public health service, the imputation of bias may be cast at him, and the old excuse and challenge of the prescribing chemist "when you stop selling medicines I will stop treating patients," may be heard again in the pharmaceutical press. This question of illicit practice in medicine and surgery, which in some quarters is conducted on a large scale, and in others is only carried on in a small way—but is very widespread indeed—has long been a grievance with the medical profession and has long been recognised by it as a source of danger to the public. All authorities are agreed that the thing is wrong and should be stopped; but not all are agreed as to the position in order of wickedness and in possibility of reform which it should occupy among the methods of unqualified practice; and many do not realise the pressure which the public, and not only its lower classes, continually brings to bear upon the chemist in order to induce him to play the part of apothecary, surgeon and physician.

The President of the Local Government Board would be well advised to read through the three-column advertisement of Mr. H. A. Barker, the bonesetter, in the *Daily Mail* of May 24. It should be added that he would do well to have this extraordinary "open letter to the President of the College of Surgeons" annotated by some surgeon of acknowledged standing; for it is, on the face of it, so extremely plausible that, without expert comment, the fallacies which it contains may pass unchallenged. We have on previous occasions lamented the indifference of newspaper proprietors to any consideration of the public weal in dealing with quacks and patent medicine advertisements. If on this occasion we have seemed to err in giving further gratuitous prominence to them, it is because we have good proof how profitable this particular form of practice must be, for a three-column advertisement in the *Daily Mail* is a somewhat expensive luxury. In our issue of February 6, 1909 (p. 475) we mentioned the very wholesome provisions of the recent legislation in New Zealand on the subject of such advertisements; it is high time that something similar were done here, and we wish the General Medical Council all success in their efforts to promote the public well-being in this matter.

